# Interactions between microbial diversity and substrate chemistry determine the fate of carbon in soil

**DOI:** 10.1038/s41598-021-97942-9

**Published:** 2021-09-29

**Authors:** Nanette C. Raczka, Juan Piñeiro, Malak M. Tfaily, Rosalie K. Chu, Mary S. Lipton, Ljiljana Pasa-Tolic, Ember Morrissey, Edward Brzostek

**Affiliations:** 1 Department of Biology, West Virginia University, Morgantown, WV USA; 2 Division of Plant and Soil Sciences, West Virginia University, Morgantown, WV USA; 3 Environmental Molecular Sciences Laboratory, Richland, WA USA; 4 Department of Environmental Science, University of Arizona, Tucson, AZ USA

**Keywords:** Environmental microbiology, Environmental sciences, Ecosystem ecology

## Abstract

Microbial decomposition drives the transformation of plant-derived substrates into microbial products that form stable soil organic matter (SOM). Recent theories have posited that decomposition depends on an interaction between SOM chemistry with microbial diversity and resulting function (e.g., enzymatic capabilities, growth rates). Here, we explicitly test these theories by coupling quantitative stable isotope probing and metabolomics to track the fate of ^13^C enriched substrates that vary in chemical composition as they are assimilated by microbes and transformed into new metabolic products in soil. We found that differences in forest nutrient economies (e.g., nutrient cycling, microbial competition) led to arbuscular mycorrhizal (AM) soils harboring greater diversity of fungi and bacteria than ectomycorrhizal (ECM) soils. When incubated with ^13^C enriched substrates, substrate type drove shifts in which species were active decomposers and the abundance of metabolic products that were reduced or saturated in the highly diverse AM soils. The decomposition pathways were more static in the less diverse, ECM soil. Importantly, the majority of these shifts were driven by taxa only present in the AM soil suggesting a strong link between microbial identity and their ability to decompose and assimilate substrates. Collectively, these results highlight an important interaction between ecosystem-level processes and microbial diversity; whereby the identity and function of active decomposers impacts the composition of decomposition products that can form stable SOM.

## Introduction

Microbial decomposition is the foundation for carbon (C) and nutrient cycling in terrestrial ecosystems and the primary step in the transformation of plant-derived substrates into stable soil organic matter^1,2^ (SOM). There is a long and rich history of research that has linked differences in the chemical composition of substrates with their subsequent decomposition rates and residence time in SOM^3,4^. Recent theoretical and empirical studies, however, have posited that the rate at which substrates are transformed and the degree to which decomposition products form stable SOM are influenced by the composition and functional traits of soil microbial communities^5,6^. While these studies have been successful in explaining empirical patterns in SOM decomposition and stabilization, the role of microbes in driving these patterns has been inferred from aggregate metrics describing composition (e.g. diversity, and/or richness). This focus on aggregate measures reflects methodological limitations in quantifying taxon-specific metabolic rates in natural communities and hinders our fundamental understanding of the contribution of individual taxa to community-level decomposition processes^7,8^. Thus, linking microbial identity with function continues to represent a grand challenge in microbial ecology^9^. Meeting this challenge also has the potential to reduce uncertainty in Earth System Models that explicitly model the impacts of microbial function and traits on soil C cycling.

**Figure 1:**
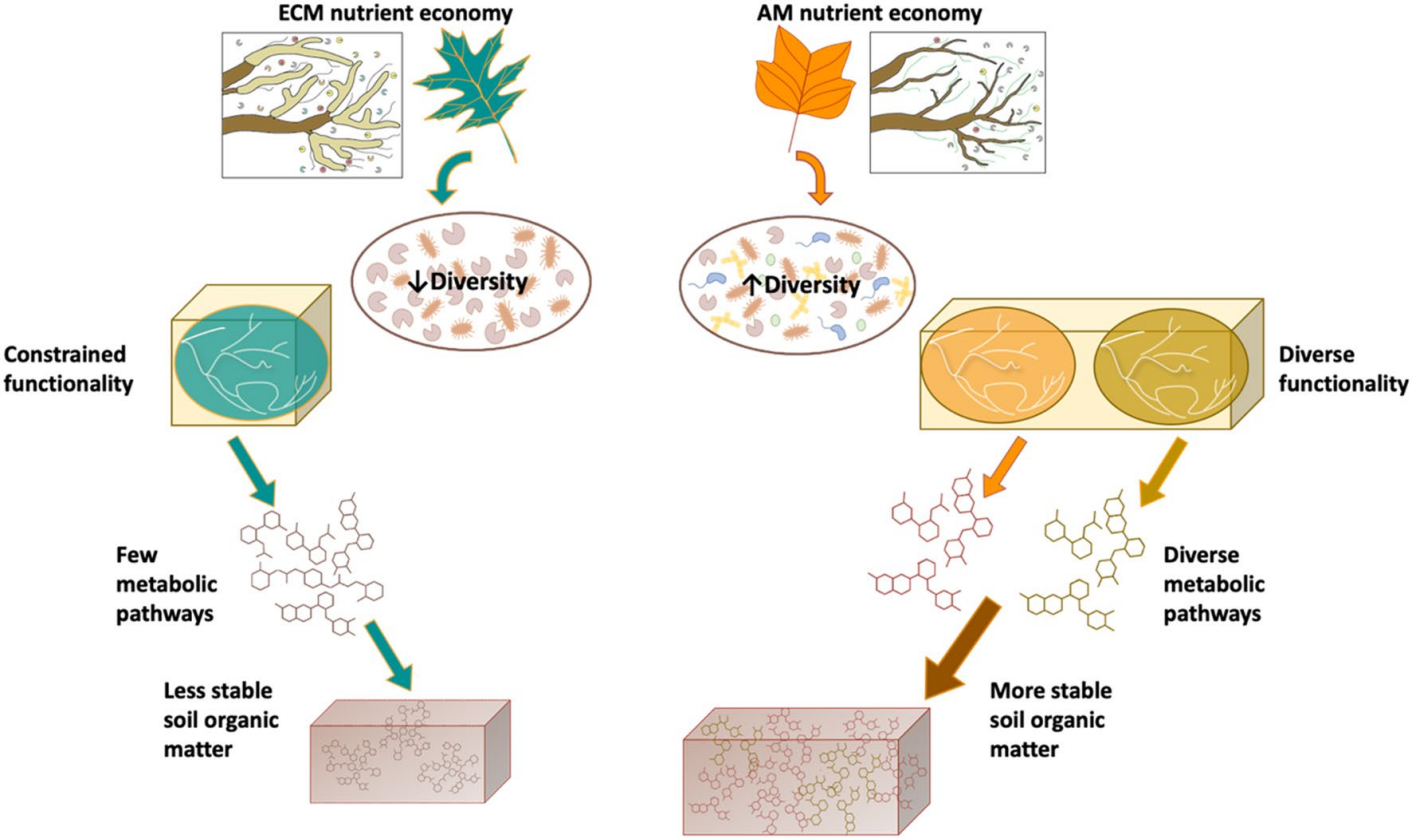
Conceptual framework of plant-microbial-soil feedbacks within ECM and AM nutrient economies. We used known differences in ECM and AM nutrient economies as the foundation for our conceptual model. ECM trees have leaf substrates that are chemically complex, which promote a low diversity microbial community that has constrained function. This constrained functionality provides only minimal metabolic pathways in which few metabolic products are produced to be sorbed onto SOM. The end result is less stable soil organic matter. In contrast, AM trees have less chemically complex substrates that can be easily degraded by most microbes. This ease of degradation may lead to a microbial community with higher diversity in both species composition and function. This diversity, in turn, generates diverse metabolic pathways that produce a wider variety of microbially processed compounds and more stable soil organic matter.

As our conceptual understanding of decomposition has evolved from theories centered on substrate chemistry to those accounting for microbial community composition and function, important interactions between these two drivers have largely been ignored. For instance, substrate and nutrient availability influence microbial biodiversity via habitat filtering and also by promoting competitive interactions between microbial guilds^7,10,11^. On the other hand, microbial biodiversity can increase soil organic matter formation through faster transformation of plant inputs into decomposition products, which can be associated to a larger number of syntrophic/facilitative biotic interactions^12–17^. We developed a new conceptual model that integrates interactions between substrate chemistry, microbial biodiversity, and the resulting decomposition products (Fig. 1). In this conceptual model, we use known differences between ecosystems dominated by trees that associate with arbuscular mycorrhizal (AM) fungi vs. those dominated by ectomycorrhizal (ECM) fungal association in their nutrient economies as our model system. ECM substrates that are chemically complex and require more energy investment to decompose (e.g. rich in lignocellulose) may foster a low diversity saprotrophic community since comparatively few species will have the metabolic capability to degrade the substrate^14–16^. In contrast, AM substrates that are less chemically complex (e.g. sugars and amino acids) can be easily decomposed by most microorganisms^18–21^ and consequently may foster more diverse saprotrophic communities. Microbial diversity can, in turn, influence the functional potential of the community to decompose a given substrate. More diverse communities in AM soils likely harbor more metabolic pathways, exhibit greater metabolic flexibility, and produce a wider variety of decomposition products that are potentially sorbed onto mineral surfaces forming stable organic matter^22^. While AM soils have been shown to have greater stable SOM pools than ECM soils across the soil profile, the cascading linkages between substrate complexity, microbial diversity, decomposition products, and stable SOM formation have yet to be investigated^23^. Moreover, our conceptual model relies on the assumption that substrates with greater chemical complexity foster less diverse microbial communities. However, there is the potential for a competing hypothesis; whereby, a chemically complex substrate heightens microbial diversity due to the need for microbes to possess a larger number of metabolic pathways to drive decomposition^24^. Thus, understanding the linkages between substrate chemistry, microbial biodiversity and the chemistry of decomposition products they produce is a critical unknown, since their characteristics may impact the rates of stable SOM formation through physio-chemical interactions with mineral surfaces^25^.

Here, we explicitly test our conceptual model (Fig. 1) using a novel approach that couples quantitative stable isotope probing^26^ (qSIP), to quantify the amount of litter C that is assimilated by active bacterial and fungal taxa, with Fourier transform ion cyclotron resonance mass spectrometry (FTICR-MS) to track changes in metabolite composition during microbial degradation. We used known differences in plant traits and microbial communities between ecosystems influenced by ECM fungi vs. AM fungi as a model system to test our conceptual framework^27,28^. ECM trees generally have leaf litter that has greater lignin content and less nitrogen than AM trees. Further, ECM trees have more reliance on rhizosphere processes to access nutrients from organic sources than AM trees^29^. ECM microbial communities have higher fungal to bacterial ratios and stronger competitive interactions between mycorrhizal symbionts and free-living microbes than AM microbial communities^30,31^. To test our conceptual model, we incubated isotopically labeled ^13^C AM (tulip poplar, *Liriodendron tulipifera*) and ECM (English oak, *Quercus rubra*) substrates in soils from AM and ECM dominated plots in a fully-factorial mesocosm experiment.

## Results and discussion

**Figure 2:**
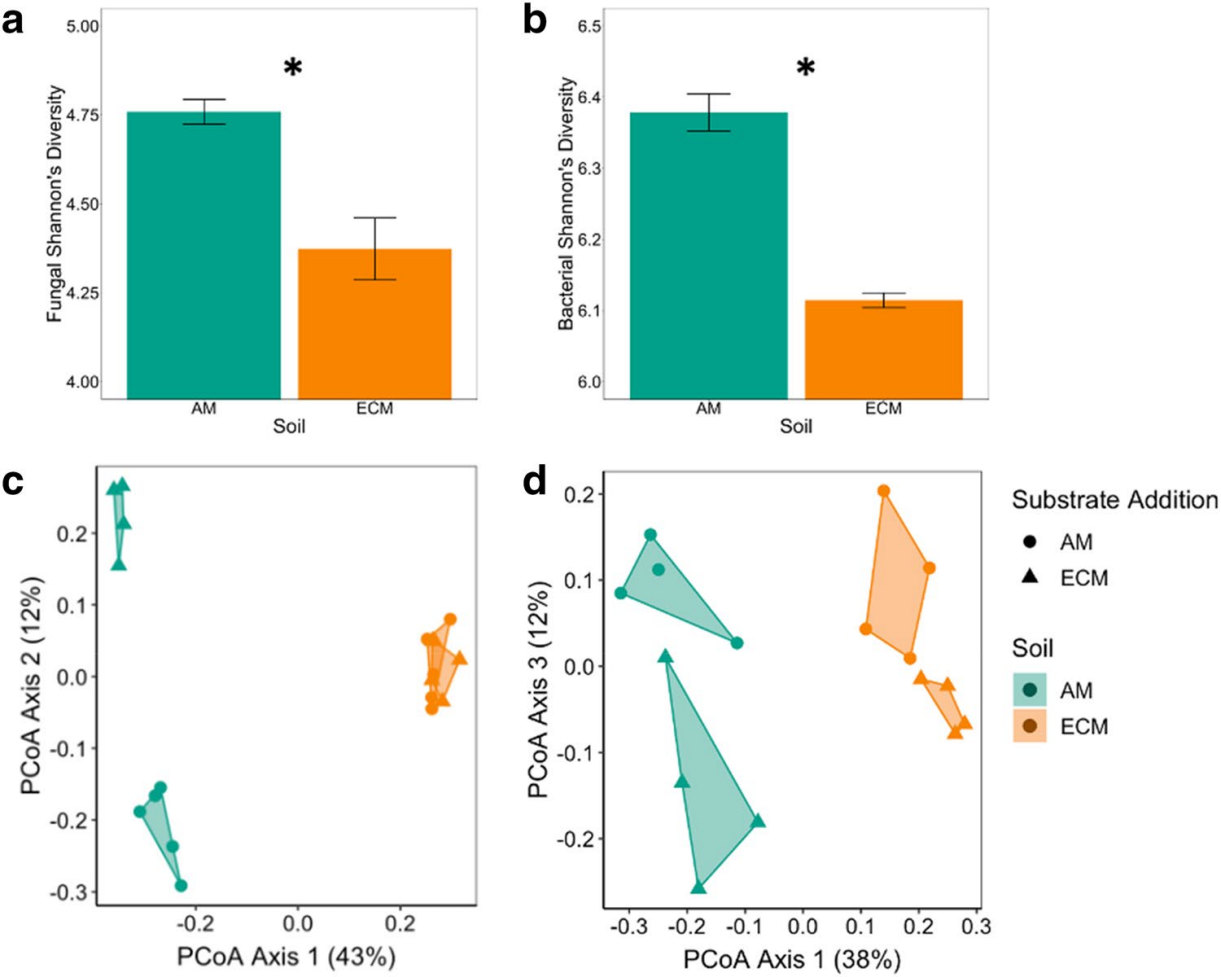
Variation in microbial diversity and composition between soils. Bar chart depicting fungal (**a**) and bacterial (**b**) Shannon diversity index (mean, standard error) in AM and ECM soils prior to incubation. Asterisks denote differences between soils at *p* < 0.05. Principal Coordinate Analysis showing variation in ^13^C substrate assimilation for fungal (**c**) and bacterial communities (**d**) as determined via quantitative stable isotope probing. For bacterial communities, there were significant differences in composition between substrates and soil type at *p* < 0.05. For fungal communities, there was a significant substrate × soil interaction at *p* < 0.05.

Here we show the extent to which differing routes of decomposition and metabolic byproducts hinge on an interaction between microbial community biodiversity with substrate chemistry. Our results support our conceptual model in that the diversity of microbes is greater in AM than ECM soils (Fig. 1) and that microbial diversity is tightly coupled to the breadth of functional capabilities and the resulting shift in SOM chemistry with microbial degradation (Fig. 2). This outcome links community ecology theory with ecosystem processes and produces a transformative framework to advance our ability to incorporate these fundamental pathways into ecosystem models.

Differences in habitat characteristics between adjacent AM and ECM-dominated tree stands over nearly 120 years of ecosystem development appear to have led to divergent microbial communities, soil chemical properties, and SOM chemical composition (Table S1, Fig. S1, S2). Fungal and bacterial communities found in AM soils were more diverse than those in ECM soils (p value < 0.01 and 0.01, respectively; Fig. 2). The greater microbial diversity in AM soils than ECM likely reflects both habitat filtering and competitive interactions. The long-term inputs of higher quality litter to AM soil likely reduced microbial stress and enhanced access to resources that fuel microbial growth and release competition^7,10,25^. By contrast, in ECM soils, the lower quality litter may have reduced diversity by selecting a lower number of taxa with the metabolic ability to degrade more complex substrates^14,16^, or by indirectly promoting greater competition for limiting resources between microbial guilds^32^. The soils also differed in C: N stoichiometry with AM soils having a lower C:N ratio (− 24%, p value < 0.05; Table S1) relative to ECM soils, a pattern commonly observed in forest ecosystems^32^. Further, microbial community composition appeared to be coupled with SOM chemical composition as determined using FTICR-MS, with AM soils possessing a different profile than ECM soils (PerMANOVA p value < 0.01, Fig. S2, Table S1). ECM soils had lower percentages of lignin- and protein-like compounds (− 43%, p value < 0.05; − 92%, p value < 0.05, respectively), but relatively more amino sugar- (+ 85%, p value < 0.05), unsaturated hydrocarbon (+ 127%, p value < 0.05) and lipid-like compounds (+ 92%, p value < 0.05) than AM soils. This lower lignin content in ECM soils may reflect a greater capacity of the saprotrophic communities in ECM soils to process lignin-rich substrates and the ability of plant-C subsidies to ECM fungi to stimulate lignin degradation^33^. Overall, these results suggest a tight coupling between differences in the nutrient economies of ECM and AM ecosystems and microbial diversity that regulate decomposition pathways to alter the chemical composition of soils.

When we followed the fate of the ^13^C AM poplar and ECM oak substrates into microbial taxa (i.e., ^13^C excess atom fraction (EAF)), we found distinctive responses to substrate type between AM and ECM microbial communities (Fig. 2, Table S2). Many bacterial and fungal taxa readily assimilated the added substrates in both soil types with the average ^13^C EAF ranging from ~ 0.04 to 0.14 for bacteria and 0.04 to 0.36 for fungi (Fig. S3). However, bacterial ^13^C assimilation was significantly different between substrates in both AM and ECM soils (substrate p value < 0.05, Table S2); whereas, fungal ^13^C assimilation was only altered by substrate type in AM soils (soil X substrate interaction p value < 0.01, Fig. 2c, d). These differences can be visualized on the principal coordinate analysis of community ^13^C assimilation (Fig. 2) which shows distinct clustering due to substrate type in the AM soil but not in the ECM soil for fungi. Importantly, AM and ECM microbial communities responded differently to substrate type even though there were similar rates of ^13^C-CO_2_ loss from the soils that was derived from the added substrate (Fig. S4). These results suggest that the greater fungal diversity in AM than ECM soils lead to communities that can rapidly shift the identity of active decomposers to metabolize plant-derived inputs of contrasting characteristics. In addition, abundance weighted ^13^C assimilation was greater in fungi than bacteria when analyzed across soils and substrate treatments (Fig. S3), supporting previous research showing that the decomposition of plant-derived substrates is driven to a greater extent by fungi than bacteria in aerobic forest soils^29,34^.

**Figure 3:**
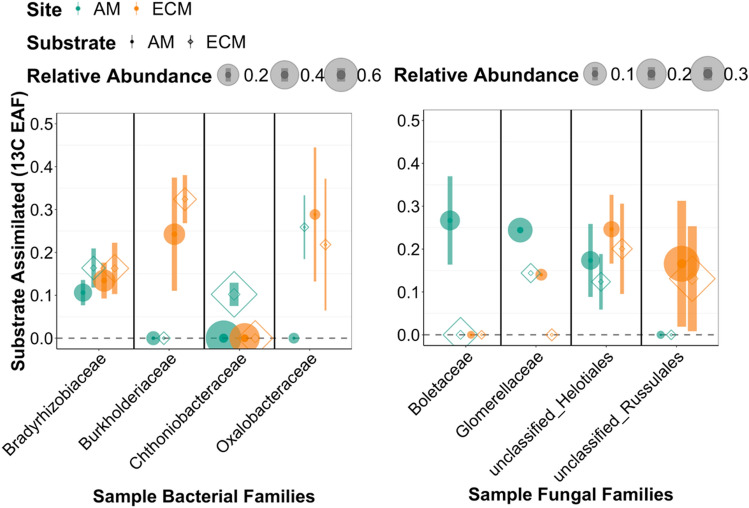
Example taxa illustrative of variation in substrate assimilation by soil and substrate type. Substrate assimilated is shown with sample bacterial and fungal families. Symbols represent averages and error bars show standard errors.

By leveraging the taxonomic resolution of our qSIP analysis, we were able to identify the fungal and bacterial families that were the dominant active decomposers out of all the taxa that assimilated the substrates across soil and substrate types (Fig. 3, S5, S6). Within fungal families, an unclassified group of *Helotiales* was an important assimilator of both substrates at both sites. Some bacterial families also appeared to be generalists assimilating both litter types in both sites (e.g. *Bradyrhizobiaceae*, *Xanthomonadaceae*, *Comamonadaceae*, and *Soilbacteraceae*)*.* However, we found that the fungal and bacterial families which assimilated most ^13^C were only present in one soil type (either AM or ECM) (Fig. S5, S6), and often showed a preference based on substrate type. For example, *Chthoniobacteraceae* were only active in the AM soil where it assimilated ECM oak but not AM poplar substrate (Fig. S5). For fungi, in AM soil, *Boletaceae* was a key assimilator of the AM poplar litter; whereas, in ECM soils, a family within *Russulales* accounted for rough 5% of C assimilation from both litter types (Fig. S6). These results highlight that decomposition of plant-derived substrates relies upon the identity of actively decomposing microbes, and that in many cases the dominant decomposing families in one system can be absent from another^34^.

We used FTICR-MS analysis to examine the extent to which differences in the composition of active saprotrophic communities between AM and ECM soils affect SOM chemical composition. In the AM soil, there were ~ threefold more unique compounds that appear as decomposition products of either AM poplar or ECM oak substrates than in the ECM soil where soils receiving AM poplar or ECM oak substrates shared a greater number of compounds (Fig. 4 a and d). These pools of molecules unique to each substrate type likely played an important role in driving the observed differences in the chemical characteristics of the broader SOM pool in AM soils. In particular, weighted mean indices of the broader SOM pool show that AM soils receiving the AM poplar substrate had greater O:C ratio (p value < 0.05), NOSC (p value < 0.001), and aromaticity values (p value < 0.05), but had lower average H:C ratio (p value < 0.05) when compared to the ECM oak substrate (Fig. 4). The ability of decomposition products to form stable SOM can be inferred through their molecular properties such as, bioavailability (i.e. higher H/C ratio), reactiveness (i.e. lower Ai) and the energetic rewards from oxidative degradation (i.e. higher NOSC)^31,35–37^. Overall, these results indicate a lower abundance of reduced and saturated C-containing compounds (i.e., the C atoms are linked by double and single bonds)^12,30,31^ and a greater abundance of compounds that require a higher energy investment to breakdown and provide a lower energy reward for microbes in the AM soils that received the AM poplar substrate when compared to ECM oak substrate. These differences are important because both metrics indicate a greater potential for the AM poplar substrate to enter the microbial stabilization loop than the ECM oak substrate in the AM soil. Higher O:C suggests more microbial processing of the AM poplar substrate in the AM soil – and because microbially processed C is more stable via organo-mineral interactions this could lead to greater stabilization interactions^12,22,38^(Fig. 4). In contrast, the ECM oak substrate has lower Ai and NOSC which should make it less susceptible to microbial decomposition due to greater activation energy.

**﻿Figure 4:**
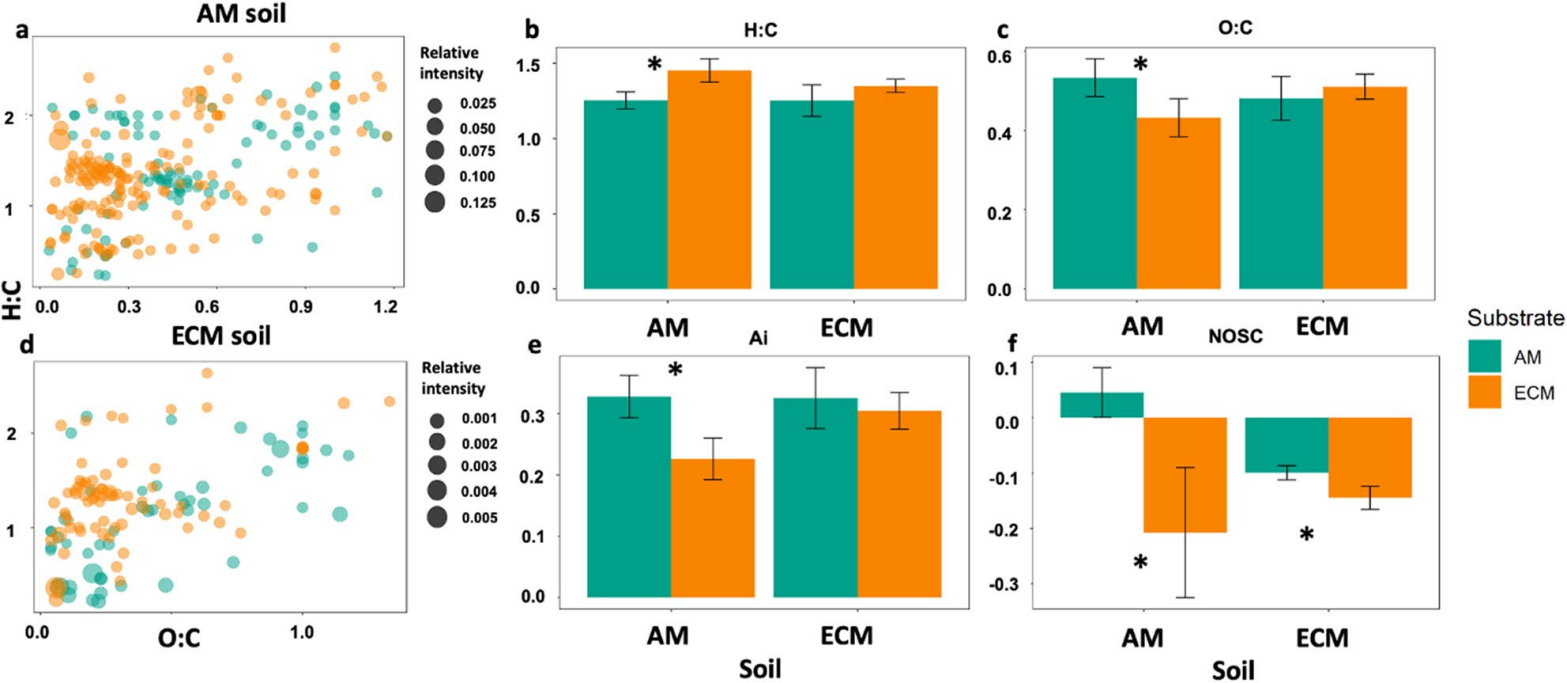
Impacts of substrate incubations on soil organic matter chemistry. Van Krevelen diagrams showing molecular formulae of compounds that are unique to each substrate addition and soil combination in (**a**) AM soil and (**d**) ECM soil. Bar plots depicting mean ± standard error of aggregated indices showing differences in the physicochemical properties, (**b**) H:C ratio, (**c**) O:C ratio, (**e**) aromaticity index (Ai), and (**f**) Nominal Oxidation State of C (NOSC), of the SOM pool. Error bars indicate standard error (n = 5). Asterisks denote differences between substrate types in each soil at *p* < 0.05.

**Figure 5:**
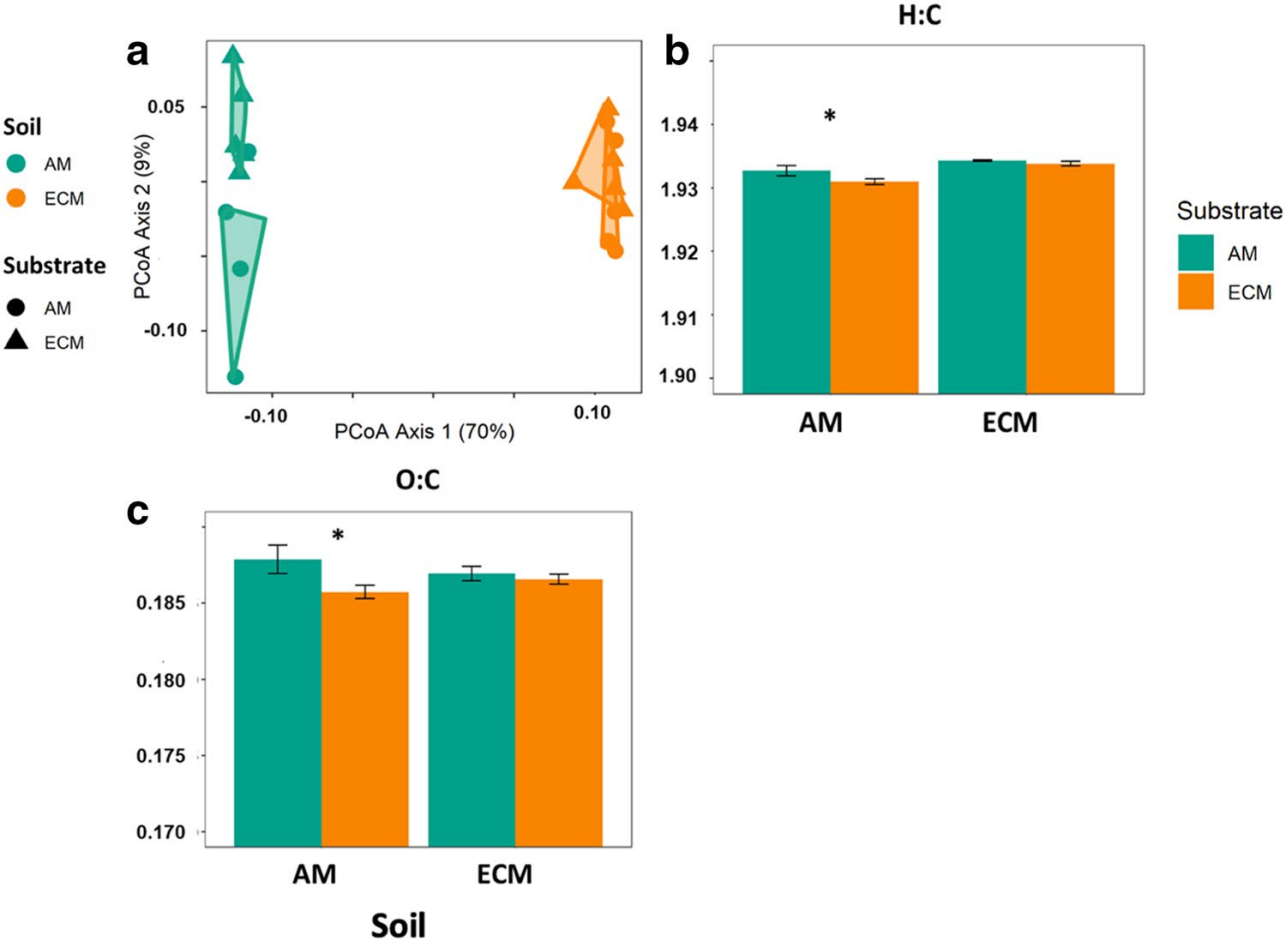
Impacts of substrate incubation on lipid pools. Principal coordinates analysis (PCoA) of the lipid pool composition in incubated soils (**a**). Bar plots depicting mean ± standard error of aggregated indices showing physicochemical properties (**b**) H:C ratio and (**c**) O:C ratio of the lipid pool. Error bars indicate standard error (n = 5). Asterisks denote differences between substrate types in each soil at *p* < 0.05.

We used lipidomics analysis to further explore the potential of decomposition products to form stable SOM, as widespread evidence suggests a dominance of microbial-originated lipids in newly-formed SOM^39^, as well as in C associated with soil minerals. Mirroring the results from the qSIP analysis, the lipidomic profiles varied with substrate type in AM soils and were unresponsive to substrate in the ECM soil (Fig. 5, Table S3). For instance, in AM soils, AM poplar substrate led to greater relative intensity of lipids of the class *Glycerophosphoethanolamines* (+ 7%, p value < 0.05), but lower intensity of *Diacylglycerols* (− 3%, p value < 0.05 Fig. S7). In addition, the trends observed on the chemical characteristics of lipids are in line with the SOM results; substrate chemistry led to significant differences in weighted mean indices of O:C and H:C ratios in AM soil but not in ECM soil (p value < 0.01; Fig. 5) . Thus, our results suggest substrate chemistry drove divergent responses in the community of active decomposers as well as their decomposition products in AM soil, leading to a distinct chemical signature that can be indicative of the microbial communities involved in the decomposition process. These results are consequential because they suggest a novel mechanistic cascade whereby the composition of active decomposers can shift in response to substrate chemistry in highly diverse microbial communities and alter the resulting decomposition products that have the potential to form stable SOM. By contrast, it appears that the active decomposers and decomposition products in soils with less diverse microbial communities are more narrowly constrained.

Collectively, these results support our conceptual model by showing a novel dynamic interplay between substrate chemistry and microbial diversity that advances our mechanistic understanding of linkages between microbial community composition and function (Fig. 1). Differences between mycorrhizal associations in their nutrient economies over the course of ecosystem development led to AM soil harboring greater microbial diversity than ECM soil (Fig. S1). This coupling of nutrient economies and microbial diversity also appears to have led to divergent functional capabilities of the resulting microbial communities. In the AM soil, greater diversity led to flexibility in the decomposition pathways with the identity of the active decomposers and the composition of their decomposition products shifting dynamically in response to variability in substrate type (Figs. 2, 3). By contrast, in the less diverse ECM soil, the decomposition pathway was comparatively static (Figs. 2, 3). The more diverse AM community generated a greater proportion of highly-processed decomposition products supporting our conceptual model that higher diversity saprotrophic communities have the potential to generate more stable soil organic matter. Moving forward, more research is needed to identify whether the patterns we observed here between AM and ECM systems operate at longer timescales (i.e., greater than 21 days) that capture slower processes that can destabilize or stabilize soil organic matter. Conceptually, these results transform our understanding of stable SOM formation by showing that diversity in microbial identity and function is coupled directly to the chemical diversity of decomposition products that are the foundation of SOM.

## Material and methods

### Soil and litter sampling

Mineral soil (0–15 cm) was collected at the Elizabeth Woods site, a 120-year-old deciduous forest in West Virginia, US (39° 32′ 50.6″ N, − 80° 00′ 00.4″ W). Soils were collected from four 20 × 20 m plots dominated by either AM-associated trees (i.e. *Liriodendron tulipifera* and *Acer saccharum*), or ECM-associated trees (i.e. *Quercus rubra*, *Quercus velutina* and *Carya ovata*). These sites have been characterized previously as Culleoka-Westmoreland silt loam soils at the AM sites and Dormont and Guernsey silt loams at the ECM sites^40^. Soils were also characterized by C:N ratios 11.7 and 14.1 for the AM and ECM soils respectively, with a pH of 6.8 for both soils. Soils with the same mycorrhizal status were pooled and homogenized, air-dried at room temperature for ~ 24 h and sieved through 2.0 mm mesh before the initiation of the experiment. Uniformly ^13^C labeled litter (> 97 atom % ^13^C) from *Quercus robur* (i.e., ECM substrate) and *Liriodendron tulipifera* (i.e. AM substrate) leaves (Isolife BV, Wageningen, NL) were incubated in soil mesocosms in a factorial design with five replicates for each treatment combination (2 soil types × 2 substrate types), along with five replicate controls (no ^13^C substrate addition) for each soil type. The ^13^C enriched substrates were dried and ground to a powder and added in a suspension of 0.5 ml sterile water to 20 g of soil at a concentration of 400 ug ^13^C g^−1^ soil. The control soils received 0.5 ml sterile water additions. These incubations were well mixed and kept at 60% water-holding capacity for the 21-day period at room-temperature^18^. Chemical characteristics of soils and plant substrates are provided in Table S1.

### DNA processing and qSIP

For quantitative stable isotope probing, DNA was extracted, quantified, ultracentrifuged, fractionated and sequenced as described in^18,26^. DNA was extracted using a MoBio PowerSoil HTP Kit following the manufacturer’s instructions. For stable isotope probing, 5 ug of DNA was loaded into a 5-ml ultracentrifuge tube with ~ 3.5 ml of a saturated cesium chloride (CsCl) solution and ~ 900 ml gradient buffer (200 mM Tris, 200 mM KCl, 2 mM EDTA). DNA was separated via ultracentrifugation at 127,000*g* for 72 h using a TLN-100 rotor in an Optima Max bench top ultracentrifuge (Beckman Coulter, Fullerton, CA, USA). Tubes were fractionated into ~ 25 fractions of 150 µl each, and the density of each fraction was measured with a Raichart AR200 digital refractometer. DNA was purified using an isopropanol precipitation method. The 16S rRNA gene was subsequently quantified and sequenced in samples containing DNA, within the density range 1.660–1.735 gml^−1^ (~ 10 fractions per sample). To quantify the 16S rRNA gene, quantitative PCR was performed in triplicate using a QuantStudio 5 applied biosystems (Thermo Fisher Scientific) and primers 515F (5′-GTGCCAGCMGCCGCGGTAA-3′) and 806R (5′-GGACTACVSGGGTATCTAAT-3′)^41^. The PCR program used was as follows: 95 °C for 2 min followed by 45 cycles of 95 °C for 30 s, 64.5 °C for 30 s and 72 °C for 1 min. Libraries were sequenced on an Illumina MiSeq instrument (Illumina, Inc., San Diego, CA, USA) using a 300-cycle v2 reagent kit. Fungal 18S rRNA gene copies in each fraction were also quantified using primers 1380F (5′-CCCTGCCHTTTGTACACAC-3′) and 1510R (5′-CCTTCYGCAGGTTCACCTAC-3′). The PCR program used was as follows: 98 °C for 3 min followed by 40 cycles of 98 °C for 45 s, 60 °C for 45 s and 72 °C for 30 s. DNA fractions were amplified for fungal ITS rRNA genes using primers ITS4F (5′-AGCCTCCGCTTATTGATATGCTTAART-3′) and 5.8SF (5′-AACTTTYRRCAAYGGATCWCT-3′)^42^ and 300-bp paired-end read chemistry on an IlluminaMiSeq (Illumina, Inc., San Diego, CA, USA). The PCR program used was as follows: 95 °C for 6 min followed by 35 cycles of 95 °C for 15 s, 55 °C for 30 s, and 72 °C for 1 min. DNA fractions were then sequenced using a 500 cycle v2 reagent kit.

Files came pre-split and joined multiple paired ends that we combined to pick operational taxonomic units (OTU). Open reference OTUs were picked at 97% identity using SILVA 128 release database for Bacteria and RDP database for Fungi. Taxa were analyzed at the ‘OTU’ level from the QIIME L7 table. Calculation of ^13^C excess atom fraction (EAF) was performed for each taxon as described previously^18,19^. Briefly, using the CsCl density gradient data, a weighted average density (WAD) was computed for each taxon’s DNA extracted from control soils that did not receive an isotopically enriched substrate. This natural abundance WAD was then compared to the taxon’s WAD following incubation with the ^13^C enriched material. The change in WAD can be used to quantify the amount of isotope incorporated into the DNA^17,18^. Preliminary data analysis revealed an effect of ultracentrifuge tube on estimation of phylotype weighted average density, probably a consequence of slight differences in CsCl density gradients between tubes. This technical error was corrected as previously described^18,19^. In addition to the samples subjected to qSIP analysis we also extracted and analyzed fungal and bacterial OTU’s from control soils where the DNA was extracted prior to incubation.

### FTICR-MS and lipidomic analyses

Soil from substrate-incubated and controls mesocosms were processed and analyzed with Fourier transform ion cyclotron resonance mass spectrometry (FTICR-MS), using a 12 T Bruker SolariX FTICR mass spectrometer at the Environmental Molecular Sciences Laboratory in Richland, WA, as described in Fudyma et al.^43^. Briefly, 100 mg of dried soil or litter substrate was extracted using an adjusted Folch extraction^44^. Extraction was performed on each sample by sequentially adding 2 ml MeOH, followed by a 5 s vortex; 4 ml CHCl_3_, followed by a 5 s vortex; sonication at 25 °C for 1 h (CPX3800 Ultrasonic Bath, Fisherbrand); addition of 1.25 ml of H_2_O, followed by a slight mix to achieve bi‐layer separation; and incubated at 4 °C overnight. The top, aqueous layer (metabolite—polar) was pipetted off into 1 ml glass vials and stored at − 80 °C until FTICR‐MS. The bottom, chloroform layer was dried down and stored in 50:50 methanol:chloroform until lipidomics analysis.

A standard Bruker electrospray ionization (ESI) source was used to generate negatively charged molecular ions in the metabolite fraction. Samples were then introduced directly to the ESI source. The instrument settings were optimized by tuning on a Suwannee River fulvic acid (SRFA) standard, purchased from International Humic Substances Society (IHCC). Blanks (HPLC grade methanol) were analyzed at the beginning and end of the day to monitor potential carry over from one sample to another. The instrument was flushed between samples using a mixture of water and methanol. One hundred and forty‐four individual scans were averaged for each sample and internally calibrated using an organic matter homologous series separated by 14 Da (CH_2_ groups). The mass measurement accuracy was less than 1 ppm for singly charged ions across a broad m/z range (m/z 300– 800). Data analysis software (Bruker Daltonik version 4.2) was used to convert raw spectra to a list of *m/z* values, applying the FTMS peak picker module with a signal-to noise ratio (S/N) threshold set to 7 and absolute intensity threshold set to the default value of 100. Chemical formulae were then assigned using in-house software following the compound identification algorithm that was described in Tolić et al.^45^. Peaks below 200 and above 800 were dropped to select only for calibrated and assigned peaks. Chemical formulae were assigned based on the following criteria: S/N > 7 and mass measurement error < 0.5 ppm, taking into consideration the presence of C, H, O, N, S, and P and excluding other elements. Detected peaks and the associated molecular formula were uploaded to the in-house pipeline FTICR R Exploratory Data Analysis (FREDA) to obtain abundance of compound classes (carbohydrate-, lipid-, protein-, amino-sugar-, lignin-, tannin-, condensed hydrocarbon-, and unsaturated hydrocarbon-like) based on molar H:C and O:C ratios of the compounds^30^. For further analysis, we only consider those masses that meet the above criteria and were detected in more than five samples. Mass-to-charge ratios with assigned molecular formulae meeting the criteria (1546 different *m/z* values) were normalized to the sum of intensities. Ions with m/z > 800 were not detected in our samples. The *m/z* values represent the molecular mass (in Dalton) of the detected ions since all detected ions were singly charged ions. While our results do not represent a quantitative characterization of OM, the values presented are relative differences and should be representative of the samples. Finally, we would like to acknowledge that we were not able to see any clear evidence of ^13^C label in our FTICR-MS analysis of the soil samples. The lack of ^13^C label in our FTICR-MS analysis of the soil samples even though they received labeled substrate could be either due to the fact that most of the labeled substrates produced by microbial activities were of low molecular weight, which cannot be detected by FTICR-MS and/or the leftover labeled substrate was of low abundance compared to the organic compounds previously present in the soil matrix. As such, we used the FTCIR-MS data to identify shifts in the overall composition of the chemical compounds in each soil.

Lipids in the chloroform fraction were analyzed by LC‐MS/MS in both positive and negative ESI modes using a linear trap quadropole (LTQ) Orbitrap Velos mass spectrometer (Thermo Fisher Scientific), as described in detail previously^46^. Lipid species were identified using the LIQUID tool^46^ followed by manual data inspection. Confidently identified lipid species were quantified using MZmine^47^ and the peak intensities were normalized by linear regression and central tendency (i.e., identifying a central or typical value for a probability distribution) using InfernoRDN.

### Statistical analysis

All data analyses were performed using R 3.2.0^48^. To examine the effects of soil type, substrate type and their interaction in the bacterial, fungal and chemical composition of DOM and the lipid pool; Bray–Curtis distance matrices were compared with permutational multivariate analysis of variance (PerMANOVA) and visualized with Principle Coordinate Analysis (PCoA) using *vegan* package^49^. PerMANOVA analysis were run on the relative abundance and on the ^13^C EAF of individual microbial taxa, separately for both bacterial and fungal communities.

The analyses for FTICR-MS were performed separately for control and incubated soils using all assigned molecular formulae remaining after quality filtering^31^. In all cases, we applied a Z-score standardization before calculating Bray–Curtis distance matrices^49^. We analyzed the results from FTICR-MS as resulting from the decomposition of the added substrates for two reasons. First, this is a fully factorial design where individual soil samples were split to either receive AM poplar or ECM oak litter substrate. Thus, each soil sample starts with the same characteristics and the changes at the end of the incubation period should reflect the processing of litter. Second, we excluded molecular formulae present in the litters and thus, the differences we report in each soil type are derived from this processing (or the lack of it).

We calculated aggregated indices that characterize both the composition and the physicochemical properties of the microbial (both bacteria and fungi) and the SOM and lipid pool^34,36^. For bacterial and fungal communities, we quantified Shannon–Weaver diversity index for each sample H′ = $-{\sum }_{i=1}^{S} pi ln(pi)$ (where *pi* is the proportion of species I) using the relative abundance of individual microbial taxa^50^. To find the percent of substrate assimilation by individual taxa, we calculated the proportion of C assimilated by each group as previously described^18,51^ as a percent. For SOM and lipid molecular formulae, we separately calculated weighted means of formula-based characteristics (i.e. *m/z*, Aromaticity Index—AI; H/C, O/C, and Nominal Oxidation State of Carbon-NOSC) as the sum of the product of the single-formula information (i.e. m/z_i_, AI_i_, H/C_i_ and NOSC_i_) and the relative intensity (I_i_) divided by the sum of all intensities (e.g., *m/z*_sample1_ = ${\sum }_{i=1}^{S}$(*m/z*_i_ ·I_i_)/Σ(I_i_)). With these metrics we obtained sample-level information related to the molecular size (i.e. *m/z*), the molecular bioavailability (i.e. higher H/C ratio), the molecular reactiveness (i.e. lower AI) and the energetic rewards from molecular oxidative degradation (i.e. higher NOSC) of the SOM, which allows to infer the potential of decomposition products to form stable SOM^12,31,35^. Detailed information of the calculated indices can be found in the literature^31,35,36^.

We further tested the effects of soil type, substrate type and their interaction on each index using the “lm” function from the “stats” package. In these analyses, P values were approximated by an F test using Type II ANOVA tests with Kenward-Roger Degrees of Freedom^52^. When interactions between soil and substrate type were found at P < 0.1, we examined differences for each level of a given factor by pairwise comparisons using the “lsmeans” package. All analyses were checked for the assumptions of residual normality and variance homogeneity.

## Supplementary Information

Supplementary Information.
