# Supplementary material for: Interactions between microbial diversity and substrate chemistry determine the fate of carbon in soil
Source: Sci Rep. 2021 Sep 29;11:19320. doi: 10.1038/s41598-021-97942-9 (PMC8481224; doi:10.1038/s41598-021-97942-9)

# Supplementary information for

**Interactions between microbial diversity and substrate chemistry determine the fate of carbon in soil**

Table of Contents

**Supplementary Tables**

**Table S1.** Soil properties

**Table S2.** Summary of PERMANOVA analysis on the effects of substrate type on fungi and bacteria community composition and ^13^C assimilation (EAF)

**Table S3.** Summary of PERMANOVA analysis on the effects of substrate type on SOM chemistry and lipids

**Supplementary Figures**

**Figure S1.** Pie chart showing the relative abundance of bacterial and fungal communities in AM and ECM soils

**Figure S2.** Pie chart showing the relative intensity of SOM chemistry groups in control soils and in added substrate

**Figure S3.** Box plots depicting minimum and maximum values of weighted average EAF for microbial type in soils

**Figure S4.** Box plots depicting the amount of ^13^C AM and ECM substrate remaining in each soil after the 21-day incubation.

**Figure S5.** Active decomposers at only one and at both sites within bacterial families in soils

**Figure S6.** Active decomposers at only one and at both sites within fungal families in soils

**Figure S7.** Bar plots depicting mean values of relative intensity of different lipid classes in AM and ECM soils in response to substrate type

# Table S1. Soil properties

| **Variable** | | **Soil** | |
| --- | --- | --- | --- |
|  |  | AM | ECM |
| C:N | | 11^±^0.141 | 14^±^0.248 |
| pH | | 6.5^±^0.150 | 6.5^±^0.133 |
| **SOM Chemistry** | |  |  |
| Amino-Sugar | | 0.03^±^0.005^a^ | 0.011^±^0.004^b^ |
| Carbohydrates | | 0.109^±^0.018 | 0.126^±^0.301 |
| Condensed HC | | 0.187^±^0.131 | 0.091^±^0.031 |
| Lignin | | 0.242^±^0.091^a^ | 0.375^±^0.092^b^ |
| Lipids | | 0.304^±^0.057^a^ | 0.115^±^0.114^b^ |
| Other | | 0.016^±^0.032 | 0.095^±^0.005 |
| Protein | | 0.037^±^0.042^a^ | 0.102^±^0.009^b^ |
| Tannin | | 0.051^±^0.021 | 0.086^±^0.021 |
| Unsaturated HC | | 0.018^±^0.003^a^ | 0.004^±^0.001^b^ |

**Table S2.** Summary of PERMANOVA analysis on the effects of substrate type on fungi and bacteria community composition and ^13^C assimilation (EAF). Bold font indicates significance at P<0.05.

**
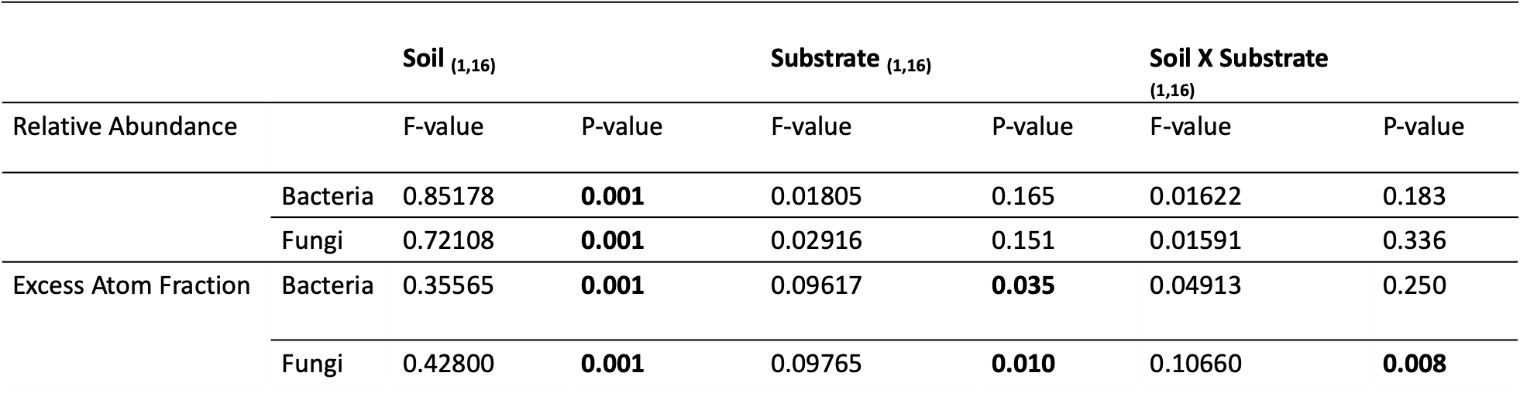
**

**Table S3.** Summary of PERMANOVA analysis on the effects of substrate type on SOM chemistry and lipids. Bold font indicates significance at P<0.05.

|  | **Soil** | | **Substrate** | | **Soil x Substrate** | |
| --- | --- | --- | --- | --- | --- | --- |
| **Variable** | **Pseudo-F_(,16)_** | **P-value** | **Pseudo-F_(,16)_** | **P-value** | **Pseudo-F_(,16)_** | **P-value** |
| **SOM Chemistry** | 2.651 | **0.039** | 1.8890 | 0.213 | 1.349 | 0.296 |
| **Lipids** | 5.781 | **0.003** | 1.951 | 0.207 | 2.867 | **0.031** |

**Figure S1.** Pie chart showing the relative abundance of bacterial and fungal communities in AM and ECM soils (n=5).


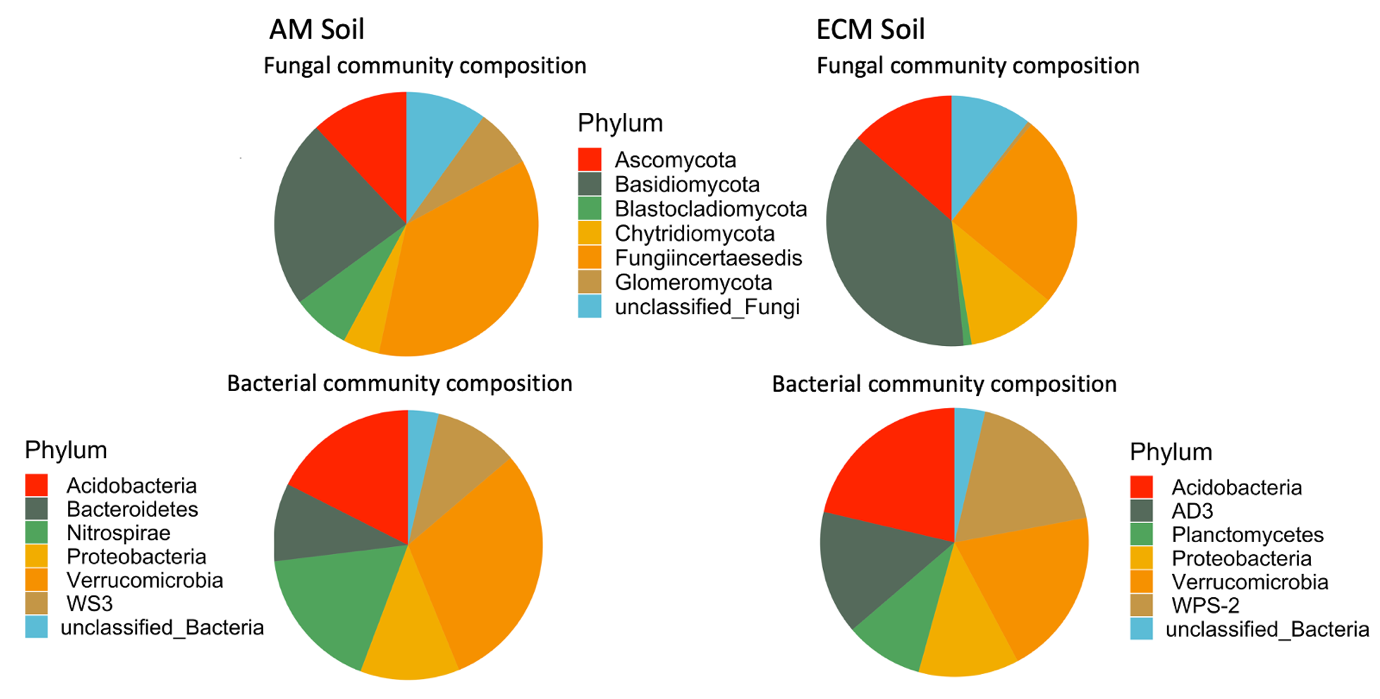


**Figure S2.** Pie chart showing the relative intensity of SOM chemistry groups in control (a) AM and (b) ECM soils (n=5) and in the added (c) AM poplar and (d) ECM oak substrate.


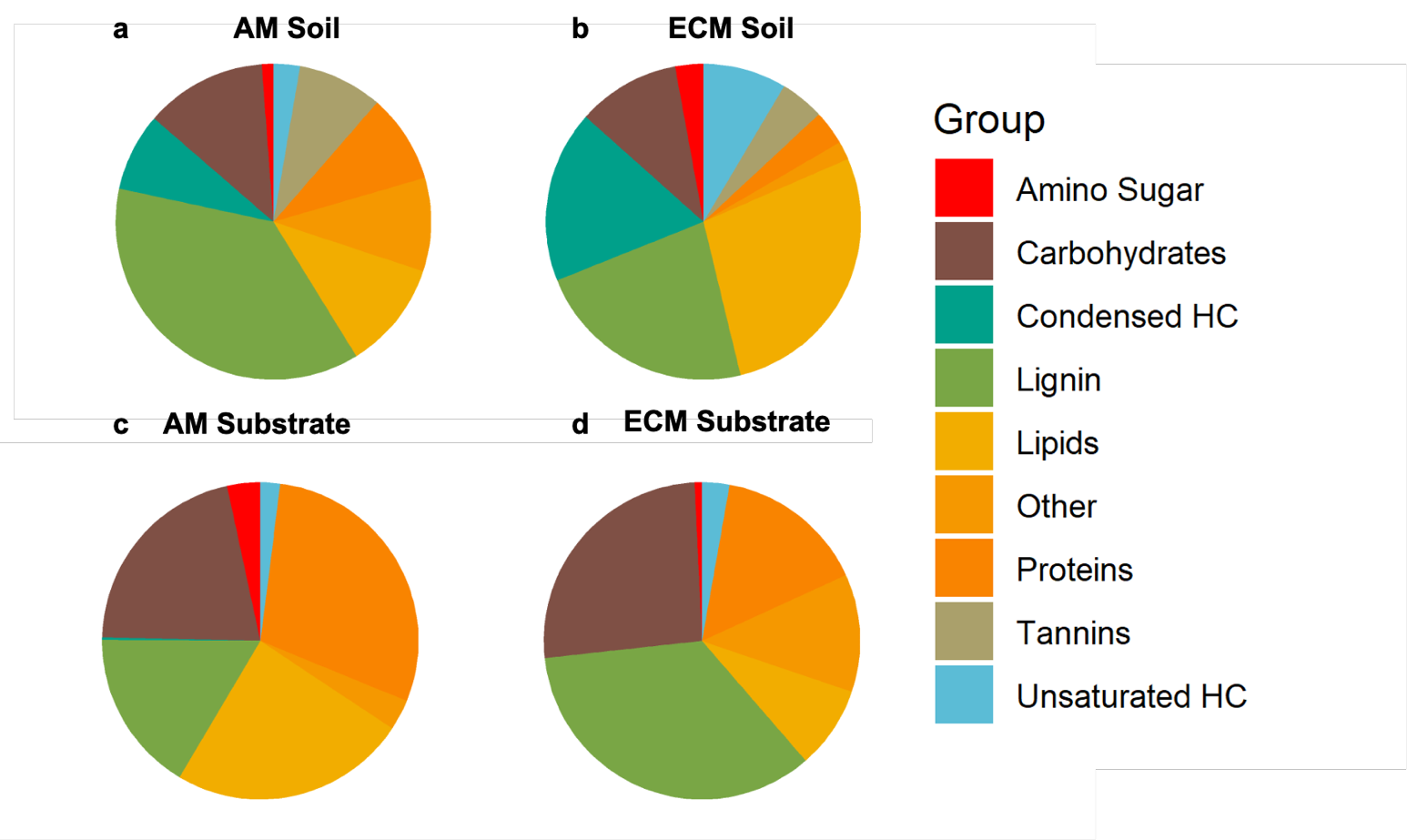


**Figure S3.** Box plots depicting minimum and maximum values of weighted average EAF for microbial type in (a) ECM and (b) AM soils in response to substrate type. Cross bars indicate median (n=5). Asterisks denote differences between bacteria and fungi at *p* < 0.05.


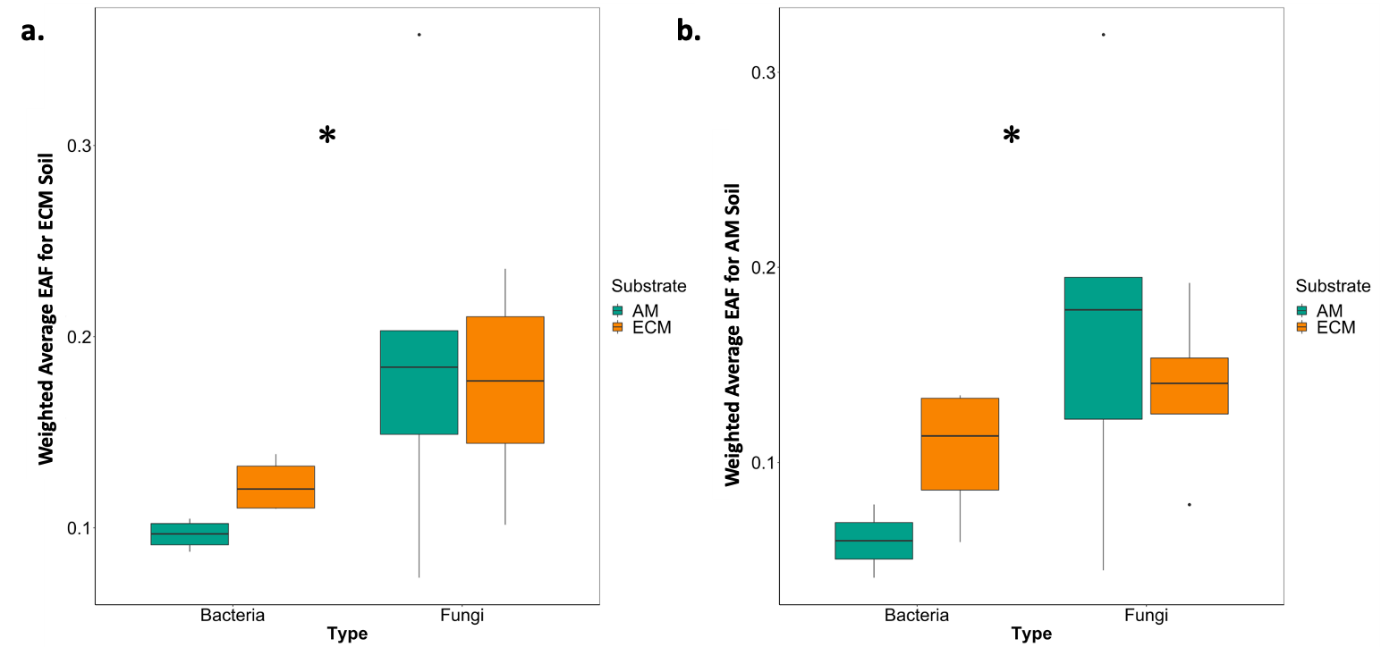


**Figure S4.** Box plots depicting the amount of ^13^C AM and ECM substrate remaining in each soil after the 21-day incubation. The ^13^C remaining was calculated by taking the amount of cumulative ^13^C respiration over the initial amount of ^13^C substrate added per gram dry weight of soil. Cross bars indicate median (n=5).


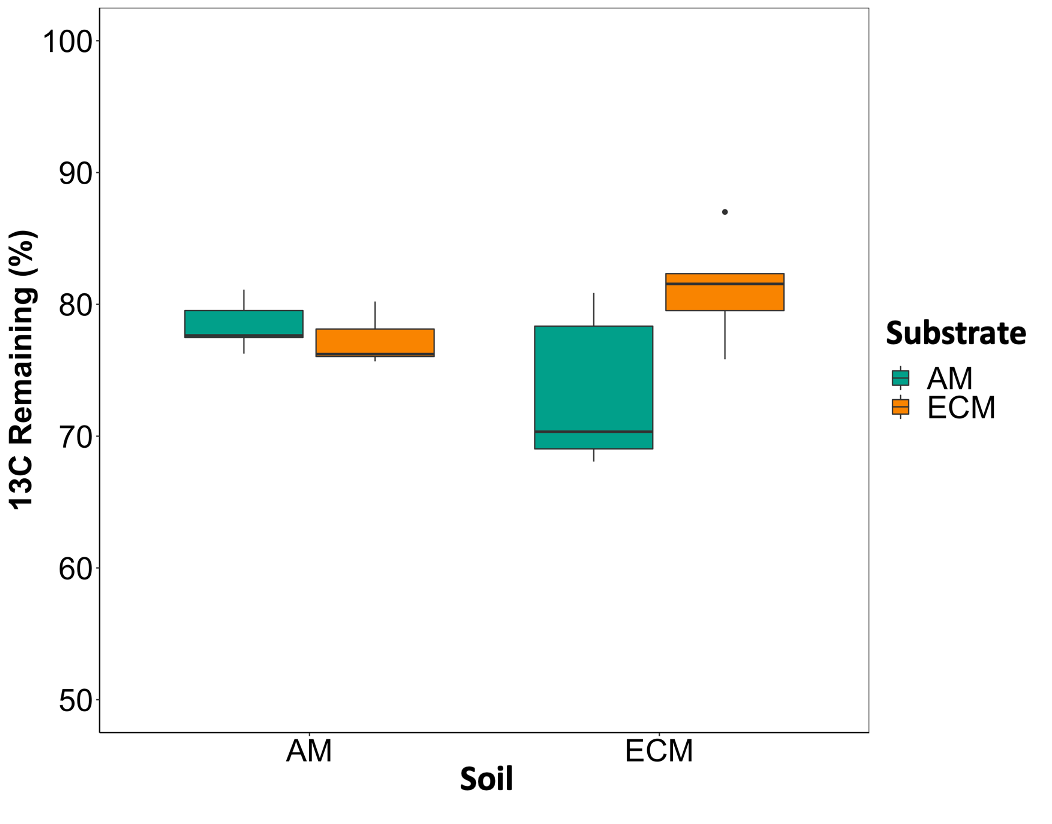


**Figure S5.** (a) Active decomposers at only one and at both sites within bacterial families in the AM soil and (b) Bacterial families at the ECM soil. Change in color denotes EAF, symbol the litter substrate and the site of the point denotes relative abundance. Error bars indicate standard error (n=5).


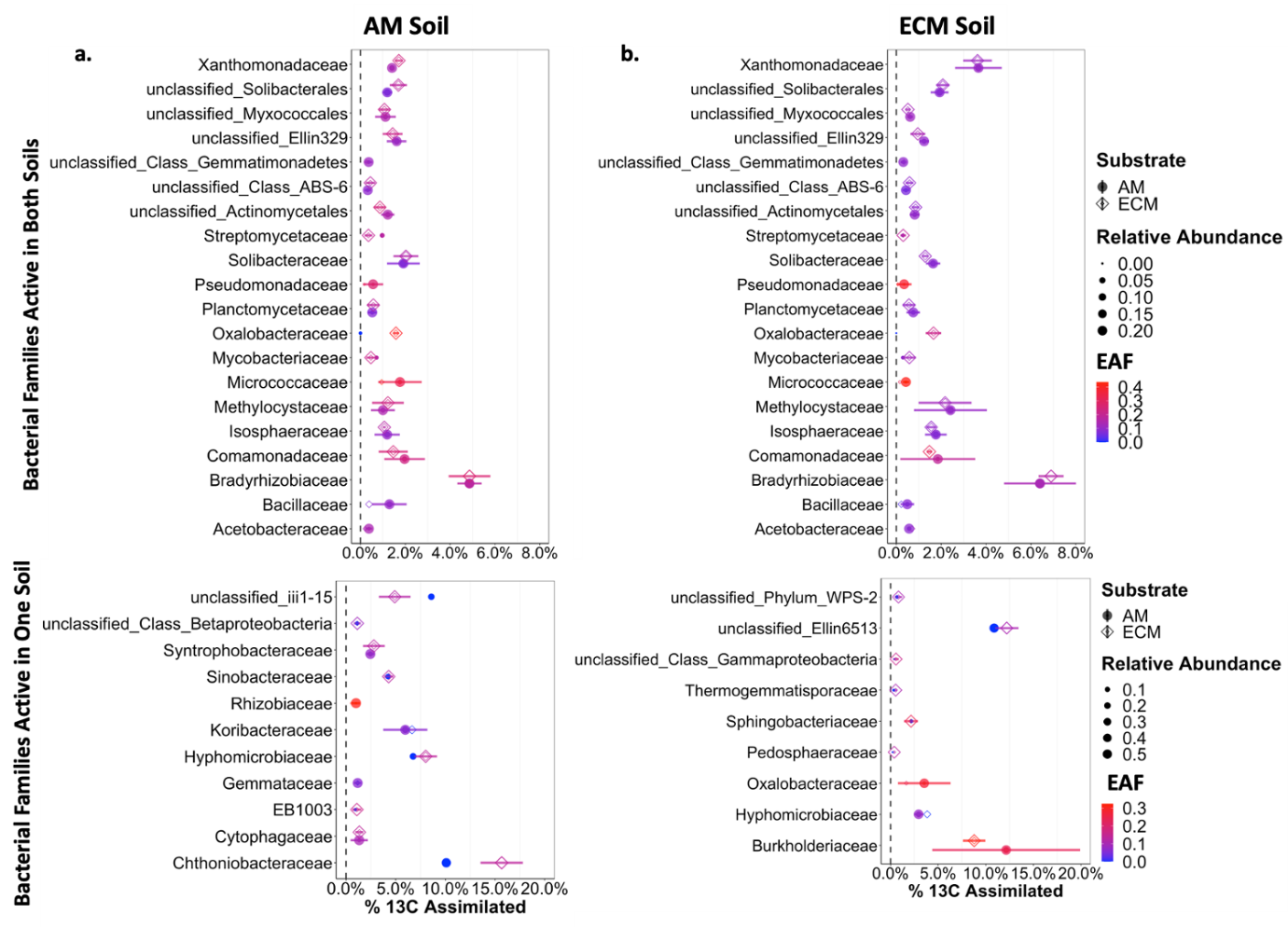


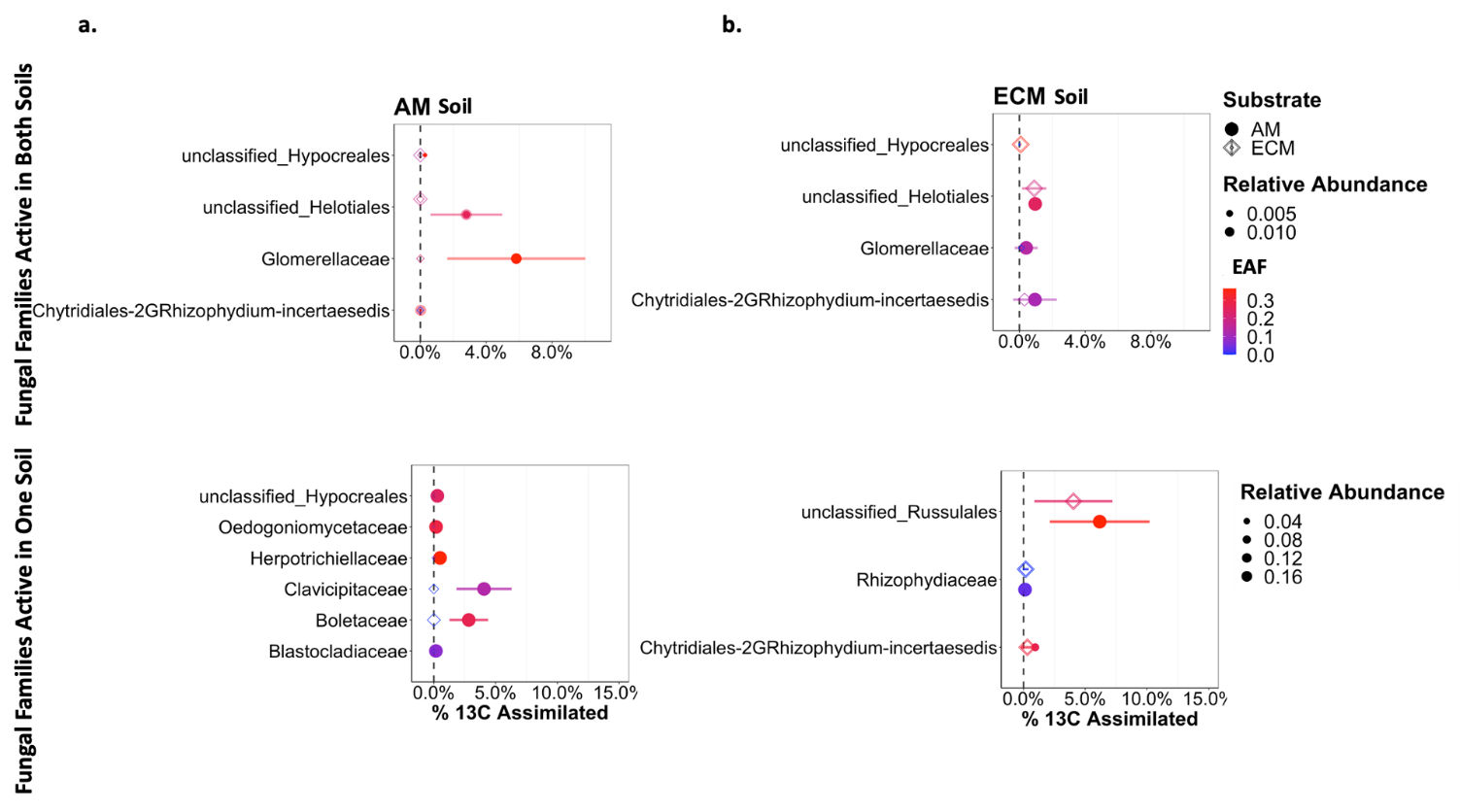
**Figure S6**. (a) Active decomposers at only one and at both sites within fungal families in the AM soil and (b) Fungal families at the ECM soil. Change in color denotes EAF, symbol the litter substrate and the site of the point denotes relative abundance. Error bars indicate standard error (n=5).

**Figure S7.** Bar plots depicting mean values of relative intensity of different lipid classes in AM and ECM soils in response to substrate type. Error bars indicate standard error (n=5). Asterisks denote differences in intensity between substrate type in each soil at *p* < 0.05. GL: Diacylglycerols Unk, GL0201: Diacylglycerols, GL0301: Triacylglycerols, GP0101: Diacylglycerophosphocholines, GP0105: Monoacylglycerophosphocholines, GP0201: Diacylglycerophosphoethanolamines, GP0202: 1-alkyl,2-acylglycerophosphoethanolamines, GP0203: 1-(1Z-alkenyl),2-acylglycerophosphoethanolamines, GP0401: Diacylglycerophosphoglycerols, GP1201: Diacylglycerophosphoglycerophosphodiradylglycerols, PR0201: Ubiquinones


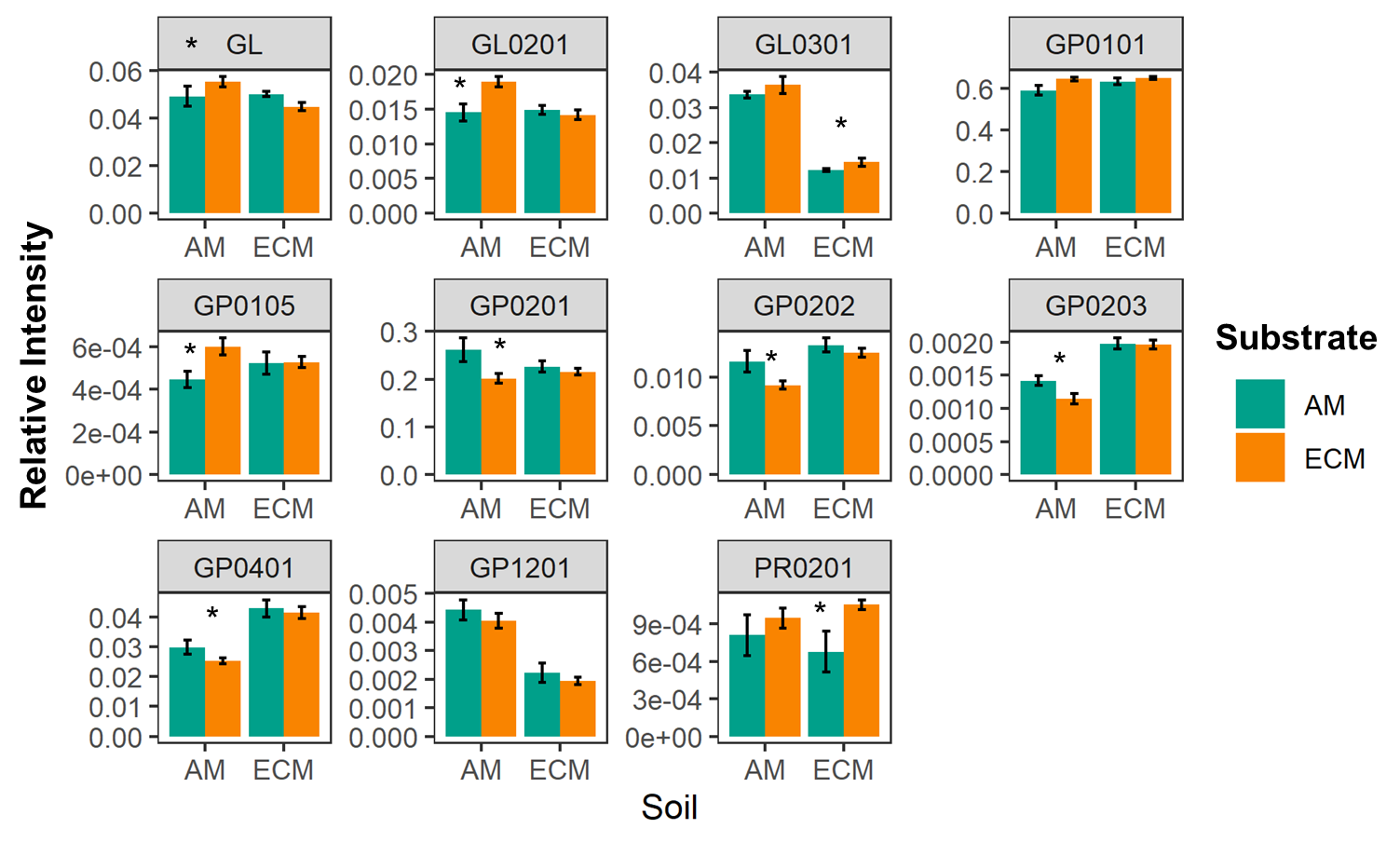

Supplement: Supplementary file 1 — Supplementary Information. [file 41598_2021_97942_MOESM1_ESM.docx]
